# Compositional Genome Contexts Affect Gene Expression Control in Sea Urchin Embryo

**DOI:** 10.1371/journal.pone.0004025

**Published:** 2008-12-29

**Authors:** Abdullah Al Mahmud, Gabriele Amore, Giorgio Bernardi

**Affiliations:** Stazione Zoologica “Anton Dohrn” Napoli, Villa Comunale, Napoli, Italy; Ecole Normale Supérieure de Lyon, France

## Abstract

Gene expression is widely perceived as exclusively controlled by the information contained in *cis*-regulatory regions. These are built in a modular way, each module being a cluster of binding sites for the transcription factors that control the level, the location and the time at which gene transcription takes place. On the other hand, results from our laboratory have shown that gene expression is affected by the compositional properties (GC levels) of the isochores in which genes are embedded, i.e. the genome context. To clarify how compositional genomic properties affect the way *cis*-regulatory information is utilized, we have changed the genome context of a GFP-reporter gene containing the complete *cis*-regulatory region of the gene *spdeadringer* (*spdri*), expressed during sea urchin embryogenesis. We have observed that GC levels higher or lower than those found in the natural genome context can alter the reporter expression pattern. We explain this as the result of an interference with the functionality of specific modules in the gene's *cis*-regulatory region. From these observations we derive the notion that the compositional properties of the genome context can affect *cis*-regulatory control of gene expression. Therefore although the way a gene works depends on the information contained in its *cis*-regulatory region, availability of such information depends on the compositional properties of the genomic context.

## Introduction

Investigations carried out in our laboratory many years ago established that genomes of vertebrates (and other eukaryotes) are mosaics of isochores, megabase-sized DNA regions, that are compositionally fairly homogeneous and belong in a small number of families covering a broad GC (the molar ratio of guanine and cytosine) range [1], [2]. This compositional compartmentalization is correlated with a number of both structural and functional properties (chromatin structure, genes and repeats distribution, introns and UTRs size; gene expression levels, replication timing, recombination). The compositional correlation between coding and flanking sequences established the concept of genome as an integrated ensemble and rejected the widely accepted view of genes being distributed at random in non-coding “junk” DNA [3]. The functional relevance of these observations was demonstrated by the fact that stable integration and appropriate expression of mammalian retroviruses was possible only in host genomic contexts of similar composition (isopycnic localization; [4], [5], [6], [7], [8]) namely when the compositional correlations that host genes have with their genome context is fulfilled [1]. Regional genomic properties are therefore relevant to the functionality of genes.

On the other hand, single gene-level studies have shown how gene expression is controlled through the utilization of the information contained inside *cis*-regulatory regions. These are composed of modules, clusters of binding sites for the factors that regulate transcription. Transcriptional control relies on the conditional activation of factors so to respond to the need of activating certain genes at specific times and in certain cells and to transcribe them at appropriate levels [9].

In the present study, we investigated if and how the compositional properties of the DNA surrounding the *cis*-regulatory region of a gene (the genome context) can affect its mode of work. We have utilized the *Strongylocentrotus purpuratus* sea urchin embryo, where we have monitored the effects of altering the compositional properties of the genome context on the expression pattern of a green fluorescent protein (GFP) reporter containing the complete *cis*-regulatory region of the *spdri* gene [10]. Activation of *spdri* happens first (from 13 to 24 h of development) in the primary mesenchyme cells (PMCs, the cells that build the embryonic skeleton). Afterward (from the onset of gastrulation) expression of the gene switches to the oral ectoderm (OE) where it is maintained throughout development. We have recently isolated a genomic DNA fragment (“4.7IL”: −3456/+389) that can faithfully replicate the gene's expression pattern. This has been verified in experiments in which this DNA fragment is fused together with a GFP coding sequence, the resulting construct (4.7IL-GFP) is injected in sea urchin zygotes and the expression pattern is observed in the developing embryos. In these experiments, 4.7IL-GFP is expressed in PMCs from 13 to 24 h and in the oral ectoderm afterward, with essentially no ectopic expression.

**Figure 1:**
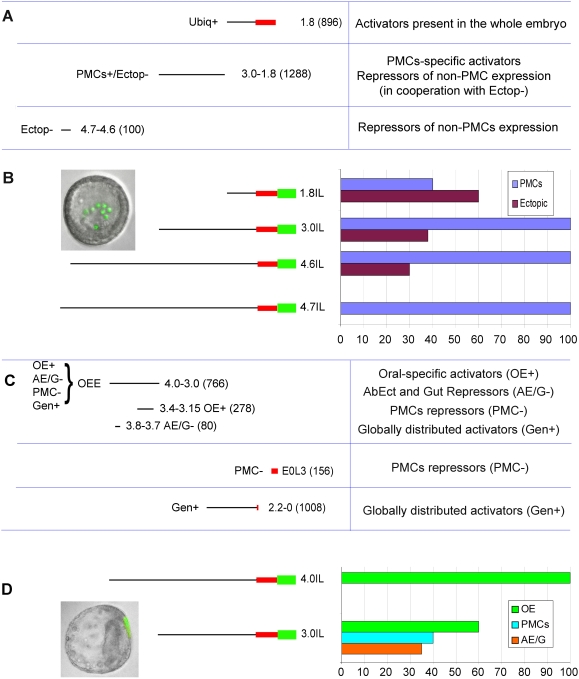
Summary of *spdri*'s *cis*-regulatory region [7]. All constructs are depicted with a black horizontal line, which represents genomic DNA, and a red box, which is *spdri*'s first exon. Constructs 4.7IL-GFP, 4.6IL-GFP, 4.0IL-GFP, 3.0IL-GFP and 1.8IL-GFP were obtained by fusing a GFP coding cassette downstream of the first exon of *spdri* (GFP cassette is indicated with a green box in the diagrams); the function of the OEE module was studied by cloning it directly into the EpGFP vector. To test the function of the E0L3L fragment, a version of 4.7IL-GFP where this element was deleted was produced [10]. Abbreviations are as follows. OE: oral ectoderm; AE: aboral ectoderm; G: gut. (A) A table showing (on the left) the PMCs modules. Here, the name of each module is given according to the function assigned (on the left of each module). Numbers on the right of each module are used to indicate the position of the extremities of the module with respect to the transcriptional start site. The size in nucleotides of each module is given in parenthesis. On the right part of the table, a brief description of the regulators operating on each module is provided. (B) A picture of a live 24 h embryo with GFP fluorescent PMCs (green cells) is shown on the left. The vegetal pole is at the bottom. The expression pattern of the indicated constructs is reported as observed in 24 h live embryos. At this stage, GFP is seen in PMCs (blue bars), at ectopic locations (purple bars), or both. (C) Regulatory modules responsible for oral ectoderm expression are shown similarly to (A). The four functions assigned to the OEE are indicated. OE+: oral specific activation; AE/G−: repression of expression in aboral ectoderm and gut; PMC−: repression of expression in PMCs; Gen+: activation in non oral territories. The position of AE/G− and OE+ is explicitly indicated. (D) A picture of a live 48 h embryo with GFP fluorescence in the oral ectoderm is show on the left. Oral ectoderm is on the right and the embryo is shown from the side. Invaginated gut is visible in its length. The vegetal pole is at the bottom. The expression pattern of the indicated constructs is reported as observed in 48 h live embryos; the consequence of removing the OEE module from 4.0IL construct is illustrated. Expression in the different territories is indicated according to the legend provided.

While a detailed description of the structure and mode of work of this *cis*-regulatory region can be found elsewhere [7], a summary of it is given in Fig. 1, where a dissection of 4.7IL is provided with a description of the function of the regulatory modules identified in it. Expression in the PMCs is obtained through the activity of three modules. A proximal module (Ubiq+; 1.8; fig. 1A) that responds to factors present in all the cells of the embryo; when this module is tested alone (construct 1.8IL-GFP; fig. 1B), GFP expression is observed in PMCs as well as in all other embryonic territories, with no bias toward a specific one. A second module (PMC+/Ectop−; 3.0−1.8; Fig. 1A) is controlled by PMCs-specific activators and by non-PMCs repressors (used to prevent ectopic expression). Addition of this module to 1.8IL results in construct 3.0IL (Fig. 1B), which drives GFP expression in PMCs of about 100% of the embryos, but maintains some ectopic expression (30% to 40%). Finally a distal module (Ectop−; 4.7−4.6; fig 1.A), is needed to completely eliminate ectopic expression, through the binding of transcriptional repressors present in cells other than PMCs. When this module is removed from 4.7IL (and construct 4.6IL is obtained; fig. 1B) the percentage of embryos expressing in the PMCs is maintained to about 100%, but the number of embryos expressing at ectopic locations remains significant (about 30%). It is likely that this module and the 3.0−1.8 module interact to ensure complete elimination of ectopic expression.

At 48 h, the 4.0IL construct (833 nt shorter at its 5′-terminus than 4.7IL; fig. 1C) ensures correct expression in the oral ectoderm (Fig. 1D). This is obtained through the “OE+” activity of the Oral Ectoderm Enhancer (OEE; fig 1C), which responds to oral ectoderm-specific activators. This module can promote transcription in the oral ectoderm after 24 h, if cloned alone into a Ep-GFP vector, which carries a sea urchin basal promoter [11]. When the OEE is removed from 4.0IL (and construct 3.0IL is obtained; fig. 1C), oral expression drops from100% to 60%. The OEE also binds repressors that are required to terminate expression in PMCs after 24 h (“PMC−” activity). This repression is obtained through a cooperation with the activity localized at the 3′ portion of *spdri*'s first exon (“E0L3”; fig. 1C). The OEE is also needed to prevent ectopic expression in embryonic aboral ectoderm and gut (“AE/G−” activity), as deletion of it increases expression in these territories conspicuously. Finally the OEE possess a general enhancer activity (Gen+); this is responsible for the expression of the reporter gene in non-oral ectoderm territories and becomes evident when the repressor portions of the module are removed. A similar “Gen+” activity is shown by the 3.0IL construct at this time; this activity is localized in the most proximal part of this genomic DNA fragment (the 2.0-0 fragment).

In the present work we have utilized this information to interpret the results of experiments in which we changed the composition of the genome context of construct 4.7IL-GFP, so that its GC level was different than that of the endogenous *spdri* gene's genomic context. As this kind of manipulation resulted in striking alteration of the expression pattern of our construct, we derived an indication that the compositional properties of the genome context can affect the way the information contained inside a *cis*-regulatory region is utilized.

## Results

### Construct molecules intersperse with carrier during concatemer formation

The *spdri*'s *cis*-regulation model presented in the previous section is based on the result of transgenesis experiments performed by microinjection. In a typical experiment, constructs are introduced in sea urchin zygotes together with restriction enzyme-digested genomic DNA, which is used as carrier (in the following referred as whole genomic DNA, WGD). It is well documented that in these conditions a construct-carrier concatemer is produced by the ligases present in the egg, which integrates in the genome after a few cell divisions and contains several hundred molecules of construct. It is assumed that in the conditions used in a standard transgenesis experiment, construct molecules are interspersed with the carrier fragments in the concatemer and that the spacing between each construct molecules depends on the molar ratio carrier: construct [12].

**Figure 2:**
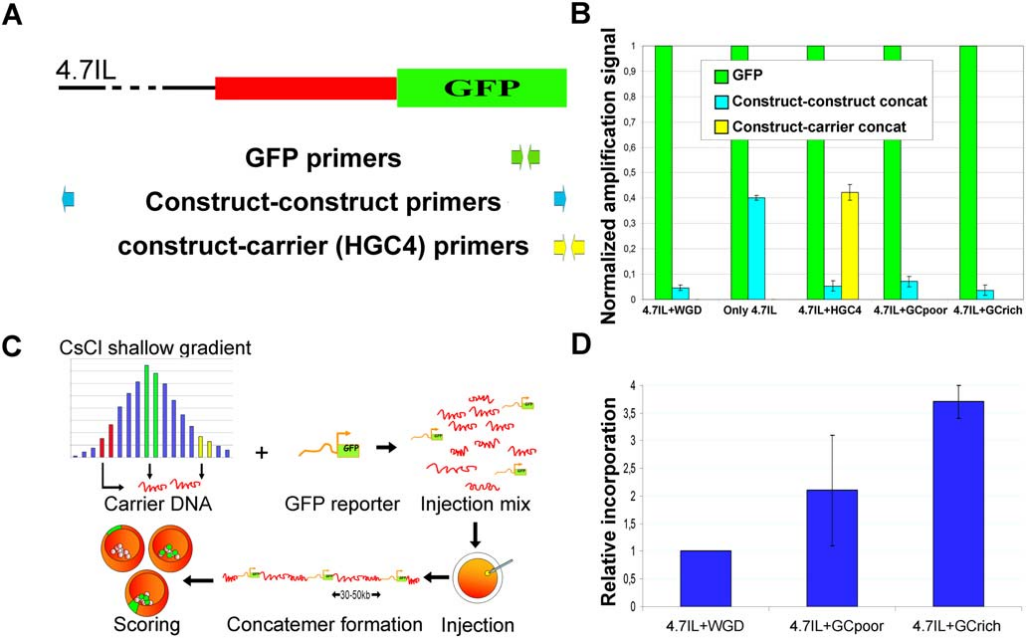
Integrated construct molecules intersperse within the concatemer. (A) A schematic view of construct 4.7IL-GFP showing the upstream portion, the first exon (red) and the GFP coding sequence (green). The portion between the upstream regulatory modules and the first exon is shown with a dashed line (not in scale). Primer couples used for quantitative PCR are shown below: in green primers used to amplify the GFP coding sequence: these two primers will only give an amplification signal if a construct-construct concatenate forms; in light blue those utilized to amplify at the junction between two adjacent construct molecules; in pale yellow those utilized to amplify at the junction between construct and carrier (HGC4 in the figure) molecules. The distance between primers is maintained around 120 nt. Carrier molecule is not shown. (B) Construct-construct (or construct-carrier) concatenation is measured Vs construct incorporation. The amount of construct molecules is measured by the level of the amplification of GFP. Construct incorporation level is equated to 1 in each experiment and the level of concatenation is measured by comparing the Ct value obtained for the amplification of construct-construct (or construct-carrier) amplification to that of construct incorporation. This allows to asses how many times concatenation occurs per each construct molecule incorporated in the genome. About 10% of the incorporated construct molecules concatenate when the WGD, a specific sequence (HGC4) or the DNA from shallow gradient fractions is used as carrier. In each experiment 100–150 embryos are utilized. Experiments were repeated using at least two different batches of embryos. (C) In order to prepare carrier DNA of chosen GC level, *S. purpuratus* genomic DNA was extracted and fractionated by CsCl shallow gradient ultracentrifugation. DNA from fractions at average GC (37.9%; green bars), GC-poor (34–35%; red bars) or GC-rich (about 40%; yellow bars) portions was utilized as carrier, in experiments where the 4.7IL-GFP construct was injected in zygotes. Upon concatemer formation, incorporation of the construct in the genomic DNA is obtained in a defined compositional context. (D) Incorporation of 4.7IL construct molecules is equal or higher when “GC-poor” or “GC-rich” carrier DNA is used compared to WGD. All the results presented here were verified in at least three separate experiments where independent batches of embryos were utilized.

Because it was critical for the correct interpretation of the data presented in this report, we sought to verify that incorporation in fact happened so that our construct molecules (4.7Il-GFP) would be evenly interspersed, within the concatemer with no significant occurrence of construct “concatenation” (*i.e.* the formation construct-construct tandems). To this aim we analysed the genomic DNA of transgenic embryos and compared the amplification signal obtained by using a couple of primers specific for the GFP coding sequence with that of a couple of primers amplifying the “junction” that would form between two concatenated construct molecules, by way of real-time quantitative PCR (qPCR). This comparison allowed us to determine how many concatenation events would occur *per* incorporated construct molecule. The results we obtained are shown in Fig. 2.

When injected together with whole genomic DNA (4.7IL+WGD in Figure 2B), the construct-construct concatenation amplification signal amounted to 4–5% of the number of integrated construct molecules. Because each construct molecule can concatenate in two different orientations with respect to the next one, this means that in a standard transgenesis experiment 10% of construct molecule concatenate at the most. On the other hand when no carrier was used (the amount of construct molecules was in this case adjusted to reach the mass of DNA necessary to achieve incorporation), the occurrence of concatenates would increase about ten times (40–45%). Therefore in these conditions almost all construct molecules (up to 90%) concatenated. When a specific sequence (HGC4 in Figure 2B, or others not shown here) would be used as carrier, construct concatenation level would be the same as that of 4.7IL+WGD. In this case we could also measure construct-carrier concatenation and observe that it was at such a level that almost all construct molecules would be flanked by carrier molecules (again about 90%, considering two possible orientations for the carrier molecules). Therefore when carrier is utilized, this allows construct molecules interspersion within the concatemer and very low occurrence of construct concatenation is observed. More importantly, the GC level of the genome context of our construct molecules is necessarily that of the DNA carrier utilized.

Compared to other genomes such as those of vertebrates, the sea urchin genome presents a low level of heterogeneity. This has been already reported in previous investigations [1] and it has been confirmed by the analysis of the sequence data from the Sea Urchin Genome Consortium [13]. The spread of the compositional distribution, as calculated on the basis of the sequence information, is between 34% and 40% GC if scaffolds equal to or larger than 25 kb are put in 0.5% GC bins. These scaffolds show a 1.8-fold increase in gene density with increasing GC-levels (manuscript in preparation). Most of the genomic DNA has a GC level comprised between 36 and 39% GC and the average value of the genome is 37.9%. This means that in a standard microinjection procedure, the compositional genome context of most of the construct molecules will be included in this range.

As the goal of our research was to investigate the effects of changing the GC level of the genomic context of our GFP constructs, we reasoned that we could obtain this by changing the GC level of our carrier DNA. Therefore we utilized two different DNA fractions from CsCl shallow gradient centrifugation of sea urchin sperm DNA, as carrier. These were recovered from the GC-poorer and the GC-richer portions of the gradient and their GC level was lower and higher than average GC level of sea urchin DNA (about 34–35% GC for “GC-poor” and about 40% GC for “GC-rich”; Fig. 2C). Here we show that when DNA from these fractions is used as carrier, construct-construct concatenation occurs at levels which are similar to those observed in a standard injection (Fig. 2B). Therefore construct molecules intersperse within the concatemer in these conditions. Furthermore we show that incorporation of construct molecules is not hampered by the use of such carrier. In comparison with standard injection procedure, the level of incorporation of construct molecules is similar or even higher when the “GC-poor” and “GC-rich” carrier are utilized (Fig. 2D).

### Changes in the compositional genome context affect cis-regulatory control of gene expression

**Figure 3:**
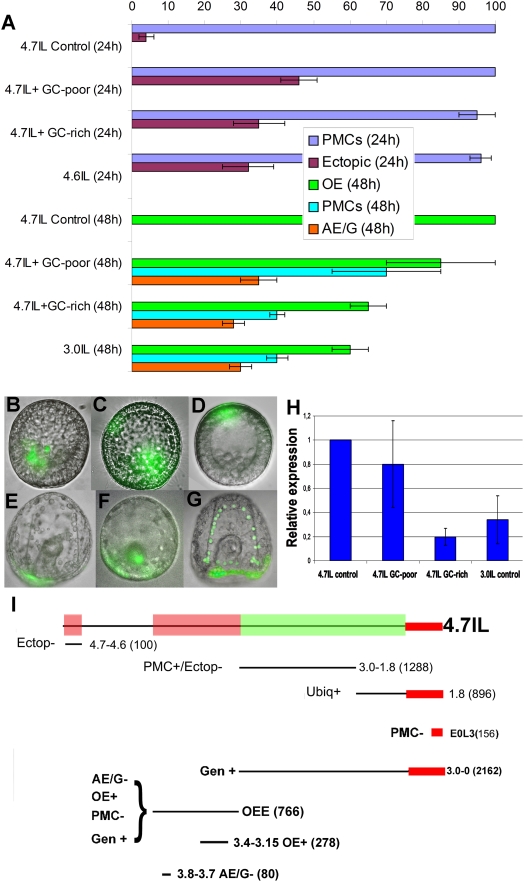
Changing the genomic context's GC level interferes with control of gene expression. (A) After injection, embryos were scored for GFP fluorescence at the times indicated in parentheses. Scoring results are given in a histogram form. Percentages of expression in the different territories are indicated according to the legend. Note that in the same embryos expression can happen in more than one territory. “Control” is used to indicate results from embryos injected with non-fractionated carrier DNA, or with carrier DNA from the central fraction of the gradient; in both cases the same result was obtained and the construct expressed appropriately. “GC-poor” and “GC-rich” indicate embryos injected with GC-poor, or GC-rich carrier DNA respectively. Abbreviations are as in fig. 1. (B–G) Representative pictures of injected embryos at 24 h (B–D) or 48 h (E–G; embryos are shown from the vegetal pole; in this view the gut appears in cross section and the PMCs form a chain around it). GFP fluorescence is present only in PMCs or oral ectoderm (B and E; control embryos), PMCs and ectopic locations (C) or in just at ectopic location (D); oral ectoderm and ectopic locations (gut in F and PMCs in G). (H) 4.7IL-GFP transcriptional output is measured at 48 h in the different injection conditions indicated. Transcriptional output measured upon injection with WGD is taken as reference and equated to 1. (I) 4.7IL-GFP is represented and aligned with its *cis*-regulatory modules. Red and green boxes over the length of 4.7IL-GFP indicate regions of the *cis*-regulatory DNA whose function is interfered with or left unaffected, upon alteration of the genome context. Abbreviations are as in Fig. 1. Note that upon injection with “GC-poor” carrier DNA the interference on the OEE function is mostly limited to its 5′ portion, where the “AE/G−” element is located.

As mentioned above, by using fractions of genomic DNA separated by CsCl shallow-gradient ultracentrifugation as a source for our carrier DNA we could embed our construct molecules in a genome context whose GC level could be manipulated. This allowed us to assess if the GC level of the genome context could affect the mode of work of our *cis*-regulatory region. In the following we present and discuss the results obtained when injecting 4.7IL-GFP in *S. purpuratus* zygotes (Fig. 3 A–I). We have also performed experiments in which we have utilized another version of the construct, D-GFP, which extends 4.7IL by 1 kb at its 5′ terminus and 897 bp at its 3′ terminus and includes the first intron and the second exon. The observations we made are consistent with those performed when injecting 4.7IL-GFP (not shown).

When injections were performed using as carrier the DNA recovered from the main fraction of the density gradient (GC level around 37.9%), the expression pattern of the injected construct was identical to that observed in a typical injection experiment (and to that of the endogenous *spdri* gene). However when fractions at the lowest and highest extremes of the compositional distribution were used, striking alterations of the expression pattern were observed. These were monitored and scored as percentages of embryos expressing GFP in the different territories at two different times: 24 h (when *spdri* is normally expressed in PMCs) and 48 h (when *spdri* is normally expressed in the oral ectoderm). The results obtained are reported in Fig. 3A. In each experiment about 100 embryos were scored and three or more different batches of embryos were used in independent experiments.

At 24 h almost all the expressing embryos correctly showed GFP-fluorescence in the PMCs. However, a conspicuous fraction of them displayed GFP-fluorescence also at ectopic locations: in 46% or in 33% of the cases (when GC-poor or GC-rich carrier DNA was used respectively) expression was observed in non-PMCs territories with no clear bias toward a specific region of the mesenchyme blastula stage embryo. This expression pattern was highly reminiscent of that observed upon injection of construct 4.6IL-GFP (Fig. 3A), which misses the distal repressor element “Ectopic−”. Therefore we interpret the observed result as if the function of this repressor module is abolished when the construct is incorporated in a GC-poor or GC-rich genomic context. As all the embryos express in PMCs, the function of the “PMC+/Ectop−” module has been maintained intact in these conditions (Fig. 3 I).

At gastrula stage, expression in the oral ectoderm was reduced from 100% of the controls, to 85% or 65% in GC-poor and GC-rich respectively. In the case GC-poor fractions were used, we observed a stronger variability in the amount of oral ectoderm expression when comparing different batches. In all the experiments expression in the aboral ectoderm and gut was observed (35% in low-GC and 28% in high-GC) as well as permanence of GFP fluorescence in PMCs (70% or more in low-GC, with high degree of variability and 40% in high-GC). These expression pattern could be compared with that of construct 3.0IL-GFP (Fig. 3 A), which lacks the entire OEE module. From this comparison it appears that in both situations (GC-poor or GC-rich genomic contexts) the function of the OEE module is interfered with (Fig. 3 I). When high-GC carrier DNA is utilized the function of the entire OEE module is abolished in a very consistent way. However when low-GC carrier DNA is utilized the extent of such interference is variable and depending on the batch the function of the OEE can be either completely or partially lost.

It is possible that the observed effects would be due to the presence of enhancers fortuitously “trapped” in the carrier DNA. Such occurrence is to be excluded based on the results of the scoring performed at 48 h. In fact if extra enhancers would be present in the carrier, these would add extra domains of expression without affecting expression in the OE, which should be always maintained in 100% of the expressing embryos. Nonetheless, to make sure that the observed behavior was not due to some non-specific effect of the carrier, or to the presence of extra enhancers, GFP vectors bearing just the basal promoter (EpGFP) were used in parallel control experiments. In this case no or minimal expression of the vector was observed. In another series of control experiments, a reporter containing the minimal enhancer “Y2Y4” from the sea urchin gene *spcyclophillin* [14] was used. The latter is a 280 bp module that only responds to two positive inputs, provided by the PMCs-specific activators *spdri* and *sp-ets1* [15] and drives expression of its GFP reporter only in these cells. When this reporter was used for control, expression could only be seen in PMCs, independently of the carrier DNA used (not shown).

Finally we measured the transcriptional output of 4.7IL at 48 h and in the different injection conditions. The results presented in Fig. 3H, clearly show that transcription was never significantly higher than that observed upon standard injection conditions. In particular the transcriptional output of 4.7IL in GC-rich contexts was similar to that of the 3.0IL construct (injected with WGD for control). Therefore we could conclude that in GC-rich context a complete interference with the OEE module was obtained. On the other hand, when the low-GC carrier DNA was used, the transcriptional output showed a greater variability (although never significantly higher than that observed with WGD). We interpret this result as if in this case the interference with the functionality of the OEE is mostly limited to its AE/G− portion (which lies at the 5′ of the OEE) and affects only marginally the functionality of the OE+ element inside the OEE module. In any case these results show that no extra enhancer was trapped in the carrier utilized.

Based on these results we conclude that the occurrence of ectopic expression, observed in the experiments with 4.7IL, was due to an interference with the functionality of specific regulatory modules inside *spdri*'s *cis*-regulatory region, not to the operation of extra enhancers present in the carrier DNA. Finally, all the effects described were not observed when the CsCl DNA fractions closer to the central part of the gradient were used. Therefore the effect observed is dependent on the GC level of the carrier DNA used.

## Discussion

The results described above give us the opportunity to sketch a picture of the relationship between *cis*-regulatory control and GC level of the genomic context. In the experiments described here, expression of our trans-gene conforms to the information contained inside its *cis*-regulatory region. However when the compositional genome context of our construct is altered, only part of this information is available and the observed alterations in the expression pattern are a direct consequence of this. Here, we have reported that a complete interference (at 24 h) with the functionality of a distal repressor module (4.7–4.6) is obtained both when GC-poor as well as GC-rich carrier DNA is used. The results obtained at 48 h show that this interference extends to the more proximal module OEE. This module seems to be completely (in GC-rich) or partially affected (in GC-poor) in the altered genomic contexts. The observed interference spreads from the most distal portions of the *cis*-regulatory region toward more proximal ones, affecting at least 1 kb of DNA (that is the distance between 4.7 and the middle of the OEE) i.e. is distance dependent. Therefore we conclude that (at least in the case described here) although the way a gene works is dependent on the information contained inside its *cis*-regulatory region, the availability of such information depends on the properties of the gene's genomic context.

Gene transcription is determined by the particular combination of transcription factors that are present at any given time and place in a cell nucleus. Upon interaction with their cognate binding sites, transcription factors mediate DNA looping so that even distant modules can be brought to interact with the basal transcriptional apparatus [9]. We hypothesize that GC level can affect the properties of genomic contexts' chromatin (condensation, methylation, nucleosome positioning), which in turn might affect the functionality of adjacent *cis*-regulatory regions. The mechanisms by which this is obtained remain to be elucidated.

In our study we have related precise alterations in the way *cis*-regulatory information is accessed to the GC level of the genome context. From our results we can derive a definition of a “proper” genome context as the DNA with compositional properties that are appropriate to allow the functionality of a gene's *cis*-regulatory region. Given the direct, measurable effect of genome context's GC level on the way *cis*-regulatory information is utilized we can imagine that changes in the genomic context's GC level can have important consequences for the functionality of genes. Such changes might result in “genomic diseases” [1], [2] where the functionality of genes might be affected even though no specific mutation in the coding or in the regulatory sequence of a gene might be identified.

## Materials and Methods

### Embryo culture, microinjection and scoring

Fertilized eggs were injected with 1–2 pl of a solution containing 250 molecules of reporter construct/pl, following described microinjection and embryo culture procedures [12]. Live embryos were observed under UV light on a Zeiss Axioscope 40; GFP expression in the different cells was assessed and embryos were scored and photographed using a Canon Powershot 6 camera.

### CsCl ultracentrifugation and carrier DNA preparation

Analytical and preparative ultracentrifugation of sea urchin genomic DNA was performed as described [16]. For preparative purposes 50 to 100 µg of genomic DNA were utilized. Fractions were eluted, UV quantified and salts were removed by drop-dialysis. To assess the GC content of individual fractions, analytical ultracentrifugation was performed with 1 µg of DNA from the fraction (when possible) or from several fractions with a higher DNA content to derive a standard curve; alternatively the GC level was derived from the refraction index of the fraction as in [2]. DNA from fractions to be used as carrier, was digested with Hind III restriction enzyme (this ensured the highest rate of incorporation). Given the carrier to construct ratio utilized in microinjection experiments, a spacing of 30–50 Kb is expected to exist between each construct molecules, upon concatemer formation and incorporation into the genomic DNA.

### Quantitative real-time PCR (qPCR) measurements

At the appropriate time point 100–150 embryos were collected and processed using the reagents in the Quiagen “All prep DNA/RNA mini kit” (Qiagen Inc., Valencia, CA). For quantitative PCR, “Fast Start Sybr Green Master” (Roche) was used. Reactions were run on an MJ Research-Biorad Chromo 4 machine, equipped with “Opticon Monitor” analysis software. Each replicate reaction was performed in a total volume of 10 or 15 µl using the equivalent amount of 2–3 embryos. For quantitative measurements an arbitrary threshold is set in the linear phase of amplification. The number of cycles necessary to reach the same amplification level (at the threshold) between different samples is converted in the corresponding ratio in the amount of DNA or cDNA. This is given by 1.94^ΔCt^ where ΔCt is the difference in the number of cycles needed to reach the threshold between samples. Therefore using transgenic embryos genomic DNA, the level of construct concatenation can be derived by comparing the amplification signal from construct-construct or construct-carrier concatenation with that of GFP coding sequence; incorporation of constructs can be assayed by comparing the GFP signal with that obtained from a single copy gene (*SpfoxA*). Transcriptional output can be estimated by comparing GFP amplification signal from cDNA between different samples after normalizing for construct incorporation and for the amount of RNA utilized (using the signal from the amplification of *Spz12* as described in [7], [14]). A baseline signal is obtained using DNA from non-injected embryos. Meaningful differences in the level of the amplification signal measured are considered those where ΔCt = >|1.7|. because of the chemistry utilized, this correspond to a time-fold change of about 3 or more.

Primers utilized for the experiments described in Fig. 2 were as follows:

Primers for construct-construct concatenation:

pGFPA: 5′-GGGAGGTGTGGGAGGTTTT-3′

q4′7r: 5′-CCTGACTGCTAAGAAAGCATTACC-3′

Primers for construct-HGC4 concatenation:

pGFPA (see above)

H4Jr: 5′-TCTTCCCGTCAACCACTTGT-3′

Primers for GFP, *SpfoxA* and *Spz12* were as in [10], [14]

### Other procedures

All other procedures utilized here are standard laboratory protocols. Detailed information on *spdri* and *spcyclophillin cis*-regulation can be found in [10], [14].
