# Correction: Compositional Genome Contexts Affect Gene Expression Control in Sea Urchin Embryo

**DOI:** 10.1371/annotation/d1eb41bd-e84d-4d53-ba71-b651f1c3058d

**Published:** 2009-01-30

**Authors:** Abdullah Al Mahmud, Gabriele Amore, Giorgio Bernardi

The Author Contributions are incorrect. They should read as follows: Conceived and designed the experiments: GA. Performed the experiments: AAM GA. Analyzed the data: AAM GA. Wrote the paper: GA GB. 

